# Deciphering bacterial mechanisms of root colonization

**DOI:** 10.1111/1758-2229.12934

**Published:** 2021-02-15

**Authors:** Hayley E. Knights, Beatriz Jorrin, Timothy L. Haskett, Philip S. Poole

**Affiliations:** ^1^ Department of Plant Sciences University of Oxford Oxford OX1 3RB UK

## Abstract

Bacterial colonization of the rhizosphere is critical for the establishment of plant–bacteria interactions that represent a key determinant of plant health and productivity. Plants influence bacterial colonization primarily through modulating the composition of their root exudates and mounting an innate immune response. The outcome is a horizontal filtering of bacteria from the surrounding soil, resulting in a gradient of reduced bacterial diversity coupled with a higher degree of bacterial specialization towards the root. Bacteria–bacteria interactions (BBIs) are also prevalent in the rhizosphere, influencing bacterial persistence and root colonization through metabolic exchanges, secretion of antimicrobial compounds and other processes. Traditionally, bacterial colonization has been examined under sterile laboratory conditions that mitigate the influence of BBIs. Using simplified synthetic bacterial communities combined with microfluidic imaging platforms and transposon mutagenesis screening approaches, we are now able to begin unravelling the molecular mechanisms at play during the early stages of root colonization. This review explores the current state of knowledge regarding bacterial root colonization and identifies key tools for future exploration.

## Introduction

**Fig. 1:**
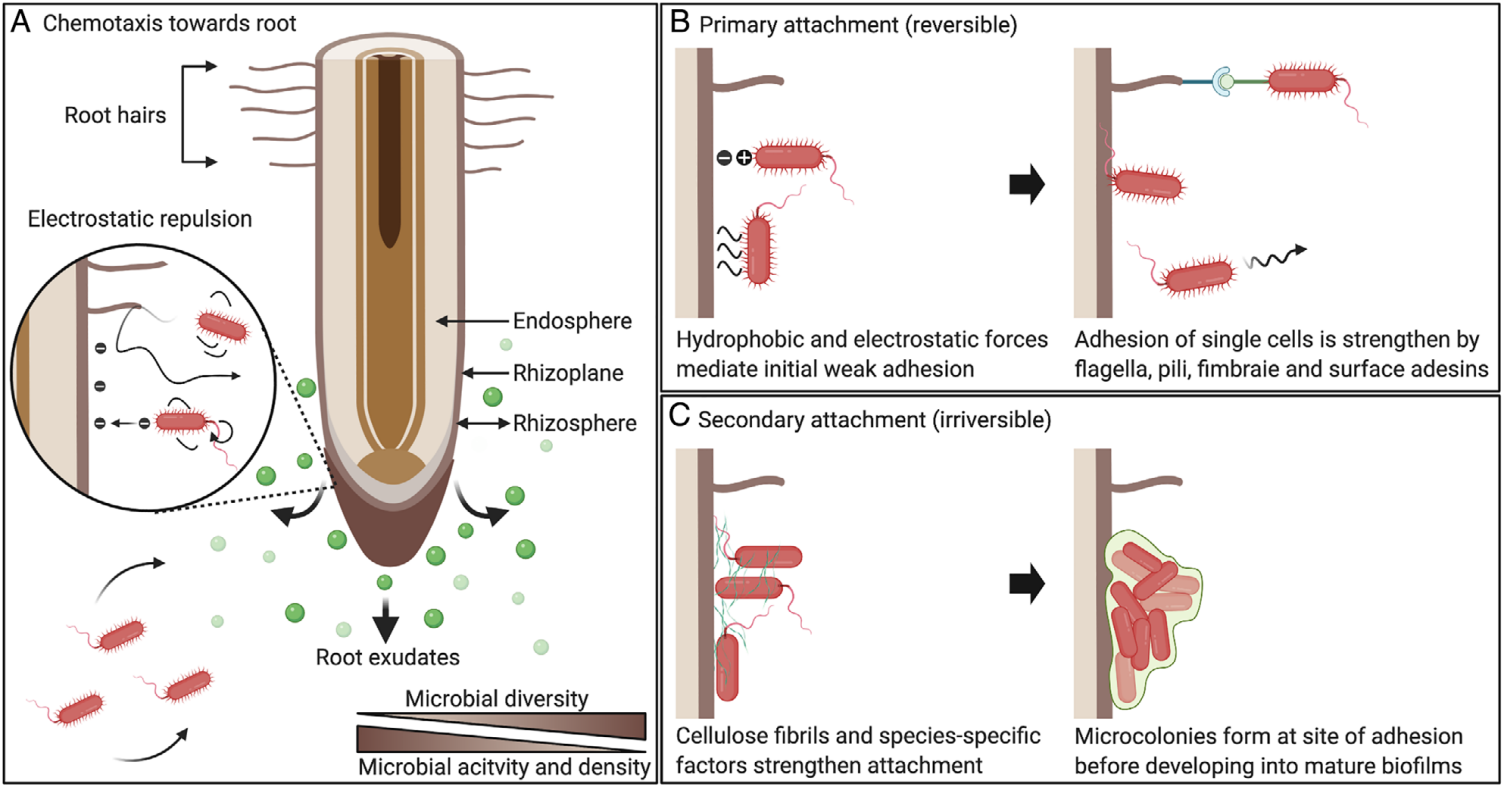
Bacterial colonization of plant roots is a multistep process. A. Plants secrete photosynthetically fixed carbon into the rhizosphere forming chemical gradients, which chemotactically attract motile bacteria from the soil towards the root surface. Flagella and pili propel bacteria, allowing them to overcome any electrostatic repulsion at the root surface. B. Primary attachment results in weak reversible binding of single cells to the root surface. This is initially mediated by hydrophobic and electrostatic interactions and subsequently strengthened by proteinaceous appendages and species‐specific surface adhesins. C. Secondary attachment leads to strong irreversible binding of bacteria to the root surface, promoting microcolony formation at the initial site of attachment. This process is mediated by the production of cellulose fibrils and other species‐specific factors including polysaccharides extracellular proteins. Created with BioRender.com

Soil provides a diverse habitat for billions of individual microorganisms, many of which form complex interactions with plants spanning the continuum of ecological outcomes from beneficial to pathogenic (Bardgett and Van Der Putten, 2014). To attract beneficial microbes from nutrient‐poor bulk soil, plants exude up to 20% of their photosynthate into the rhizosphere (soil–root interface), providing carbon for microbial growth and proliferation (Estabrook and Yoder, 1998). Some individuals form more intimate associations with plants, colonizing the rhizoplane (root surface) as epiphytes or endosphere (space between root cells) as endophytes (Fig. 1A) (Bulgarelli *et al*., 2013; Reinhold‐Hurek *et al*., 2015; Tkacz *et al*., 2015). Epiphytic and endophytic lifestyles allow microorganisms to remain anchored in a nutrient‐rich environment and facilitate the development of beneficial plant–bacteria interactions (PBIs), thus providing a key advantage over a free‐living lifestyle.

Root‐associated microbiota positively influence plant health and productivity through various mechanisms including enhancing nutrient acquisition, priming of plant defences and control of plant pathogens (Berendsen *et al*., 2012; Philippot *et al*., 2013; Wei *et al*., 2015; Trivedi *et al*., 2020). In recent years, metagenome studies have identified a wealth of microorganisms inhabiting the various root niches and revealed bacteria to be the most prevalent form of root‐associated microbiota. Despite the vast bacterial diversity present in soil, bacteria from four phyla, Actinobacteria, Bacteroidetes, Firmicutes and Proteobacteria, account for the major fraction of the root microbiome (Uroz *et al*., 2010; Bulgarelli *et al*., 2012; Lundberg *et al*., 2012). However, taxonomic composition varies widely at the genus and species levels due to unique selective pressures imposed by host genotype, sub‐localisation and abiotic environmental factors (Turner *et al*., 2013a; Turner *et al*., 2013b; Schlaeppi *et al*., 2014; Lebeis *et al*., 2015; Tkacz *et al*., 2020).

Exploitation of the root microbiome is often touted for its enormous potential to substitute environmentally deleterious agrochemicals such as fertilizers and pesticides that are crucial for current agricultural productivity (Busby *et al*., 2017). As such, there is escalating interest regarding the mechanistic characterization of beneficial PBIs. Genomics and multi‐omics approaches have facilitated the identification of many bacterial genes shared across phylogenetically diverse bacterial taxa involved in adaption to root niches, such as those required for colonization and bacteria–bacteria interactions (BBIs) (Levy *et al*., 2018). However, the processes driving bacterial assembly in root niches at the community level remain elusive since studies have predominantly validated the role of individual genes through analysis of loss‐of‐function mutations on sterile root systems. One exception is the recent application of simplified synthetic communities (SynComs) of bacteria, which have begun to shed light on the influence of individual bacteria and plant‐derived metabolites during the colonization of root niches. In this review we explore the current knowledge of bacterial root colonization from chemotaxis towards the rhizosphere, through to attachment on the root surface (Table 1) and highlight the tools available to aid future characterization of bacterial assembly in root niches.

**Table 1:** Genes shown to affect colonization of plant roots.

Colonization stage	Category	Gene(s)	Strain(s)	Comments	Reference(s)
Chemotaxis towards the root	Chemotaxis	*cheA*	*P*. *fluorescens*	*cheA* mutants demonstrate reduced competitive tomato‐root‐tip colonization when inoculated 1:1 with wild‐type	de Weert *et al*. (2002)
		*che1* gene clusters	*R*. *leguminosarum*	Che1, which encodes the conserved set of *cheAWYRB* homologues, facilitates root colonization and promotes competitive nodulation of peas	Miller *et al*. (2007)
		*mcpA*, *mcpB*, *tlpA*, *tlpB*, *mcpC*, *tlpC*, *yvaQ*, *yoaH*, *yfmS*, *heamAT*	*B*. *subtilis*	Deletion of all 10 chemoreceptors results in reduced colonization of *Arabidopsis* roots relative to wild type	Allard‐Massicotte *et al*. (2016)
		*cheY1*, *cheY2*	*A*. *caulinodans*	Regulate chemotaxis, competitive root colonization and competitive nodulation of *Sesbania rostrata*	Liu *et al*. (2020)
Movement towards/over the root and potential role in primary attachment as adhesins	Flagella	*fliC*, *fleQ*, *fleS*	*P*. *fluorescens*	Single mutants are non‐motile. Colonize alfalfa roots when inoculated alone but impaired in colonization when co‐inoculated with wild type. These genes have been found in every pseudomonad analysed	Capdevila *et al*. (2004)
		*flhD*, *flhC*	*S*. *enterica*	Double mutant is non‐motile as defective in flagella synthesis and fails to effectively colonize *Arabidopsis* roots or invade lateral root junctions	Cooley *et al*. (2003)
		*flmA*, *flmB*	*A*. *brasilense*	Mutation in either gene results in non‐motile cells due to altered flagellum assembly. Mutations also affect cells competitive ability to attach to maize roots	Rossi *et al*. (2016)
	Type IV pili	*pilA*, *pilB*	*Azoarcus* sp. BH72	Single and double mutants failed to colonize rice roots or infect the root epidermal cells	Dörr *et al*. (1998)
Primary attachment	Major outer membrane proteins (MOMPs)	*oprF*	Specific to *Pseudomonas* genus	Purified OprF strongly and selectively binds wheat, barley, maize and sunflower roots, but not leaves. *Pseudomonas fluorescens* o*prF* mutants show reduced attachment to cucumber and tomato roots 1 h post‐inoculation	De Mot and Vanderleyden (1991); Alvarez Crespo and Valverde (2009)
		*omaA*	*A*. *brasilense*	Purified OmaA has a stronger binding affinity for cereal roots relative to legume and tomato roots	Burdman *et al*. (2001)
	Unipolar polysaccharide (UPP)	*gmsA*	*R*. *leguminosarum*	Determines glucomannan synthesis which under acidic conditions mediates reversible polar attachment of single cells to pea and vetch roots by binding plant lectins	Laus *et al*. (2006)
Secondary attachment and/or microcolony and biofilm formation		*uppABCDEF*	*A*. *tumefaciens*	Encodes UPP similar to glucomannan but mediates irreversible polar attachment to plant tissue and abiotic surfaces	Tomlinson and Fuqua (2009); Xu *et al*. (2012)
	Cellulose fibrils	*celA*	*R*. *leguminosarum*	Encodes cellulose synthase. Mutation does not alter attachment to root hairs but prevents cap formation under acidic and alkaline conditions. Mutants were also able to form biofilms in vitro (on glass) but not on root hairs	Williams *et al*. (2008)
			*A*. *tumefaciens*	Mutants attach to carrot tissue culture cells but unable to form aggregates and easily removed by washing plant tissue	Matthysse (1983)
	Extracellular proteins	*lapA*	Specific to *P*. *fluorescens* *P*. *putida*	Surface adhesin and biofilm matrix component. Drives transition from reversible to irreversible attachment. *Pseudomonas putida lapA* mutants are impaired in competitive colonization of corn roots	Espinosa‐Urgel *et al*. (2000); Hinsa *et al*. (2003); Yousef‐Coronado *et al*. (2008); Gjermansen *et al*. (2010); Duque *et al*. (2013)
		*lapF*	Specific to *P*. *putida*	Mediates cell–cell interactions during biofilm development. Mutants impaired in micrology formation and biofilm development. Also impaired in individual and competitive colonization for corn and alfalfa roots	Martinez‐Gil *et al*. (2010)
		*rapA1*	Confined to *R*. *leguminosarum* and *R*. *etli*	Overexpression of *rapA1* in *R*. *leguminosarum bv*. *trifolii* increases attachment to red clover roots fivefold	Mongiardini *et al*. (2008)
Important for attachment to plant roots but not to abiotic surfaces	LPS	*rffB*	*Rhizobium* sp. IRBG74 >99% identity to *A*. *tumefaciens rffB*	Mutants impaired in dTDP‐rhamnose synthesis resulting in altered LPS. Show reduced ability to colonize legume and rice roots, which negatively affects nodulation and endophytic colonization respectively. Mutants not affected in attachment to polypropylene plates so plant attachment specific trait	Mitra *et al*. (2016)
		*rmlD*	*A*. *brasilense*	Disruption of dTDP‐rhamnose biosynthesis modifies LPS core and increased EPS production resulting in impaired attachment to maize roots and reduced colonization	Jofré *et al*. (2004)
		*rfbB*, *rfbC*	*H*. *seropedicae*	Mutants lack rhamnose‐containing LPS and show 100‐fold reduction in attachment to maize roots relative to wild type. Also impaired in endophytic colonization. No difference in attachment to glass fibre suggesting recognition of rhamnose‐containing LPS is important for colonization of hosts	Balsanelli *et al*. (2010)
Root‐hair attachment	EPS	*pssA*	*R*. *leguminosarum*	*pssA* regulates EPS biosynthesis, with mutants showing reduced attachment to root hairs and impaired cap formation. Attachment to the epidermis was still observed	Williams *et al*. (2008)

### Chemotaxis towards the root

Plant photosynthates secreted into the rhizosphere form gradients that are perceived by bacteria occupying the surrounding soil, resulting in activation of chemosensory pathways and movement of motile bacteria towards the root (Fig. 1A). Bacterial motility is primarily mediated by proteinaceous appendages termed flagella that protrude from the cell surface or through type IV pili (Alexandre, 2015). Genes homologous to known components of the chemosensory and flagella pathways have been identified in most sequenced bacterial genomes suggesting that chemotaxis provides a selective advantage, particularly in nutrient scarce environments such as soil (Armitage, 1999; Szurmant and Ordal, 2004; Wadhams and Armitage, 2004).

Not surprisingly, inactivation of bacterial chemotaxis or motility renders bacteria deficient for colonization of the rhizoplane (Ames and Bergman, 1981; Bauer and Caetano‐Anollés, 1990; de Weert *et al*., 2002; Allard‐Massicotte *et al*., 2016). This is illustrated in experiments where all the *Bacillus subtilis* chemoreceptors were genetically inactivated leading to a significant reduction in colonization of *Arabidopsis thaliana* roots 4 h post‐inoculation (Allard‐Massicotte *et al*., 2016). Chemotaxis and motility also play fundamental roles in the formation of *Rhizobium*‐legume symbioses. Rhizobia are motile alpha‐proteobacteria that infect legume root nodules and fix atmospheric di‐nitrogen (N_2_) into ammonia for plant utilization in return for carbon (Poole *et al*., 2018). Successful establishment of nodule symbiosis requires a highly specific molecular dialogue between the two partners. Beginning in the rhizosphere, exudation of chemoattractants by legumes draw rhizobia towards the root hairs which act as the entry point for nodule infection (Armitage *et al*., 1988; Barbour *et al*., 1991; Dharmatilake and Bauer, 1992). Mutation of chemosensory components impairs competitiveness for root colonization and nodule occupancy in *Rhizobium leguminosarum* (Miller *et al*., 2007) and *Azorhizobium caulinodans* (Liu *et al*., 2020). Although these mutant bacteria remain motile, they are unable to sense chemical gradients towards the root or possibly root hairs where most nodule infections are initiated.

### Root attachment

Attachment of bacteria to the rhizoplane marks the first physical step in many PBIs, anchoring bacteria in the nutrient‐rich environment of the rhizosphere and securing a prime location for the subsequent development of more intimate associations. The molecular mechanisms underlying root attachment have been best defined in the agriculturally important bacterial genera: *Rhizobium*, *Pseudomonas*, *Azospirillum*, *Agrobacterium* and *Salmonella* (Wheatley and Poole, 2018). These proteobacteria share a common biphasic mechanism consisting of two phases: primary attachment, characterized by reversible binding of bacteria to the root surface, followed by secondary attachment which results in their irreversible adhesion.

#### Primary attachment

Primary attachment involves weak, non‐specific and reversible binding mediated by hydrophobic and electrostatic interactions between cells and adjacent surface molecules on the root (van Loosdrecht *et al*., 1987; Kendall and Roberts, 2015). Despite the benefits of root attachment only a small proportion of inoculated isogenic bacteria, typically representing 0.4%–3.5% of the population, actually attach to roots in controlled conditions (Rodríguez‐Navarro *et al*., 2007). This is primarily due to electrostatic repulsion, which occurs between the negatively charged bacterial cell envelope and root surface (Berne *et al*., 2015). To overcome these repulsive forces, bacteria use flagella and pili to propel themselves towards the root surface (Fig. 1A). Following these initial interactions adhesins present on the cell surface mediate a tighter but still reversible association with the root (Wheatley and Poole, 2018) (Fig. 1B). Bacterial adhesins involved in primary attachment include proteinaceous appendages (flagella, pili, fimbriae), surface proteins and polysaccharides (exo‐ and capsular polysaccharides).

Numerous studies have demonstrated the role of flagella and pili as adhesins, enabling bacteria to not only move to the root but also to attach and migrate across the root surface. Flagella‐defective mutants of *A*. *brasilense* fail to attach to wheat or maize roots, whereas purified polar flagella bind to wheat roots (Croes *et al*., 1993; Rossi *et al*., 2016). Likewise, *P*. *fluorescens* and *S*. *enterica* flagella‐deficient mutants are unable to competitively colonize alfalfa roots or invade *Arabidopsis* lateral root junctions respectively (Cooley *et al*., 2003; Capdevila *et al*., 2004). In the plant pathogen *P*. *aeruginosa* (Hahn, 1997; O'Toole and Kolter, 1998) and N_2_ fixing endophyte *Azoarcus* sp. BH72 type IV pili act as adhesins (Dörr *et al*., 1998). However, it is often difficult to elucidate whether adhesion or motility facilitated by the flagella and pili is necessary for primary attachment. Fimbriae also take part in primary attachment, but unlike flagella and pili they do not have an active role in motility. Fimbriae appear to be common primary attachment factors among rhizobacteria (Vesper and Bauer, 1986; Vesper, 1987; Tan *et al*., 2016), which contain a high proportion of hydrophobic amino acid residues thereby contributing to cell surface hydrophobicity and influencing attachment (Rosenberg and Kjelleberg, 1986; Donlan, 2002).

Considering that bacterial mutants lacking flagella, pili and fimbriae are still able to attach to the root surface (Tan *et al*., 2016), it is likely that other species‐specific factors with adhesive properties such as polysaccharides and surface proteins play a key role in primary attachment. For example, in *A*. *brasilense* and *P*. *fluorescens* various major outer membrane proteins (MOMPs) have been implicated in root adhesion and cellular aggregation (De Mot and Vanderleyden, 1991; Burdman *et al*., 2001; Alvarez Crespo and Valverde, 2009). These MOMPs are exposed on the outer side of the bacterial cell and function by interacting with the surface domains of proteins and polysaccharides located on the root exterior.

#### Secondary attachment

The second phase of attachment involves strong irreversible binding of the bacteria to the root surface, mediated by the synthesis of extracellular cellulose fibrils and species‐specific secondary attachment factors. Biosynthesis, secretion or exposure of these cellulose fibrils and secondary attachment factors is typically induced after successful primary attachment (Matthysse, 1983; Ausmees *et al*., 1999; Martinez‐Gil *et al*., 2010; Monteiro *et al*., 2012). Secondary attachment culminates in the formation of a bacterial microcolony on the root (Fig. 1C) and ensures that bacteria remain on the rhizoplane. For many bacteria, this is essential for subsequent endophytic colonization (Kandel *et al*., 2017).

Cellulose fibrils, which often extrude from multiple points over the bacterial cell surface, appear to be universal secondary attachment factors among proteobacteria (Thompson *et al*., 2018). These fibrils bind tightly to one another, thereby promoting the formation of bacterial aggregates on the rhizoplane. In *Rhizobium*, cellulose fibrils assist bacterial accumulation at the site of infection by tightly adhering rhizobial cells on root hair tips (Smit *et al*., 1987; Williams *et al*., 2008). Similarly, in *Agrobacterium* cellulose fibrils anchor bacteria at the site of primary attachment promoting tumour formation (Matthysse, 1983). Although attachment is a critical early step in *Rhizobium* infection and *Agrobacterium* pathogenesis, the role of cellulose fibrils in attachment is not essential for the establishment of these PBIs. *Rhizobium* and *Agrobacterium* mutants deficient in cellulose fibrils are still able to induce nodulation and tumour formation respectively (Matthysse, 1983; Smit *et al*., 1987). Nonetheless, it cannot be excluded that cellulose fibrils are important for attachment and development of these PBIs under field conditions since *Agrobacterium* mutants lacking cellulose fibrils are easily removed from the rhizoplane by washing and require inoculation of higher cell densities to induce tumour formation (Minnemeyer *et al*., 1991).

In addition to the conserved factors described above, species‐specific factors play a key role in secondary attachment. These factors include extracellular proteins and polysaccharides that permit accumulation of bacteria at the site of primary attachment (Rodríguez‐Navarro *et al*., 2007). In *P*. *fluorescens* and *P*. *putida* the large adhesin protein LapA defines the transition from reversible polar attachment of single cells to their irreversible adhesion (Hinsa *et al*., 2003). LapA is a Ca^2+^ binding protein secreted from the bacterial cell through ATP binding cassette transporters, which loosely associates with the bacterial cell surface ready to mediate surface interactions. *Pseudomonas fluorescens lapA* mutants attach to abiotic surfaces at levels comparable to the wild type 1‐h post‐inoculation (hpi) but after 5‐hpi show a significant reduction in attachment and are defective for biofilm formation, suggesting that LapA is not involved in the initial primary attachment of *P*. *fluorescens* to roots. In contrast, *P*. *putida lapA* mutants show reduced attachment to abiotic surfaces and corn seeds and are defective for biofilm formation at 1‐hpi implying that LapA plays a role in the initial adhesion of *P*. *putida* (Espinosa‐Urgel *et al*., 2000; Yousef‐Coronado *et al*., 2008; Duque *et al*., 2013). Moreover, *P*. *putida lapA* mutants are at a competitive disadvantage for colonization of corn roots when in competition with the wild type strain. In *R*. *leguminosarum* and *R*. *etli*, *Rhizobium*‐adhering proteins are important species‐specific primary attachment factors also thought to bind Ca^2+^ (Ramey *et al*., 2004). RapA1 is a secreted Ca^2+^‐binding protein that localizes on the extracellular surface at the cell poles and is predicted to promote aggregation through binding of exopolysaccharides (EPS) or capsular polysaccharide (Russo *et al*., 2006). Overexpression of *rapA1* in *R*. *leguminosarum* enhances the number of bacteria attached to host legume roots by up to fivefold (Mongiardini *et al*., 2008).

Some species‐specific adhesins such as EPS and lipopolysaccharides (LPS) play a role in both the primary and secondary attachment of diverse bacterial species. EPS is a major cell surface component composed of carbohydrate polymers, which promote cellular aggregation and irreversible binding to the root surface by forming bridges between bacterial cells (Burdman *et al*., 2000). The structure of these polymers varies considerably between bacterial species, altering the electrostatic, hydrophobic and steric properties of the cell surface and in turn affecting attachment. In *R*. *leguminosarum*, mutation of the EPS biosynthesis regulator *pssA* results in reduced attachment to root hairs and impaired aggregation at root hair tips (Williams *et al*., 2008). However, attachment to the root epidermis was still observed. LPSs constitute a major component of the Gram‐negative outer membrane and are composed of large tripartite glycolipids with a hydrophobic portion called lipid A, a hydrophilic core oligosaccharide, and the hydrophilic O‐antigen side chain (Bertani and Ruiz, 2018). LPS plays a critical role in the establishment of effective associations between several plant growth–promoting bacteria and their hosts. *Rhizobium* mutants deficient in the production of the monosaccharide rhamnose, an integral component of the LPS O‐antigen, displayed reduced colonization of rice and *Sesbania rostrata* roots, nullifying plant growth promotion and impairing nodulation respectively (Mitra *et al*., 2016). Disruption of rhamnose biosynthesis also alters LPS composition in *A*. *brasilense*, *Herbaspirillum seropedicae* and *A*. *caulinodans* resulting in reduced colonization of the host plants (Jofré *et al*., 2004; Balsanelli *et al*., 2010) and for *A*. *caulinodans*, ineffective symbiosis with *S*. *rostrata* (Gao *et al*., 2001). Interestingly, several of these LPS mutants were not impaired in attachment to plastic and glass surfaces, indicating that LPS or a component of it is not required for general attachment (Balsanelli *et al*., 2010; Mitra *et al*., 2016).

#### Environmental factors influence root attachment

Attachment can be influenced by environmental factors such as soil pH, divalent cations (Ca^2+^ and Mg^2+^) and water availability (Caetano‐Anollés *et al*., 1989; Howieson *et al*., 1993). The effect of pH on attachment has been well characterized in *R*. *leguminosarum*. Under acidic conditions the unipolar polysaccharide (UPP) glucomannan mediates localized polar attachment of *R*. *leguminosarum* to pea and vetch root hairs through binding to plant lectins (Laus *et al*., 2006). The gene locus encoding glucomannan is conserved among *Rhizobium* and *Agrobacterium* where a UPP similar to glucomannan has been shown to mediate irreversible polar attachment to plant tissue (Tomlinson and Fuqua, 2009; Xu *et al*., 2012). Under alkaline conditions root lectins are solubilized preventing glucomannan from mediating attachment. It has been proposed that an extracellular Ca^2+^ binding protein termed ‘rhicadhesin’ may mediate attachment under alkaline conditions. This rhicadhesin is predicted to bind the bacterial cell wall via a Ca^2+^ that is thought to dissociate under acidic pH conditions; however, there is currently little evidence pertaining to the identity of this hypothetical protein (Matthysse, 2014; Thompson *et al*., 2018). In fact, evidence for the existence of rhicadhesin is based entirely on a single set of experiments where crude preparations of rhizobial membrane proteins inhibited attachment of various rhizobia to pea roots (Smit *et al*., 1989; Smit *et al*., 1991). Thus, it remains unclear as to whether a pure rhicadhesin protein facilitates attachment or whether the concerted action of several proteins is required for this process.

### Bacterial biofilms

Following attachment, microcolonies develop into mature biofilms on the root surface. Biofilm formation is a key determinant of successful root colonization and is a common strategy employed by many soil bacteria. Biofilms provide a physical barrier against detrimental external stimulus such as the diffusion of antimicrobial compounds from the host plant or other microbiome members. They also protect bacteria from environmental stresses including changes in pH, osmotic stress and UV radiation (Davey and O'Toole, 2000). Fundamentally, biofilms consist of dynamic heterogeneous communities of bacterial cells embedded in a matrix of EPS which aids in adherence to the root surface and ensures cells remain proximal to one another (Branda *et al*., 2005; Flemming and Wingender, 2010). Within the biofilm, individual microcolonies are separated by water channels that facilitate diffusion of nutrients, oxygen, antimicrobial compounds and even DNA via horizontal gene transfer (Donlan, 2002; Flemming and Wingender, 2010); hence biofilms also play a significant role in the functioning of BBIs. Large adhesins play a fundamental role in biofilm formation by mediating cell–cell interactions in both Gram‐negative and Gram‐positive bacteria. In *P*. *putida*, *lapF* mutants are unable to form microcolonies at the initial site of attachment and display reduced colonization of corn and alfalfa roots when inoculated individually and co‐inoculated with the wild type (Martinez‐Gil *et al*., 2010). This suggests that LapF plays a role in both root colonization and the development of mature biofilms in *P*. *putida*. LapA has also been implicated in mediating cell–cell as well as cell–surface interactions during biofilm development in *P*. *putida* (Gjermansen *et al*., 2010). No orthologues of *lapA* or *lapF* have been identified in *P*. *syringae*, *P*. *mendocina*, *P*. *stutzeri* or *P*. *aeruginosa* strains suggesting that although both pathogenic and non‐pathogenic strains of *Pseudomonas* attach to, colonize and form biofilms on surfaces, the mechanisms by which they do this differ (Duque *et al*., 2013). Other large adhesins such as Bap and Esp have been shown to mediate surface colonization and biofilm formation in the Gram‐positive bacteria *Staphylococcus* and *Enterococcus* respectively (Cucarella *et al*., 2001; Toledo‐Arana *et al*., 2001). These proteins along with other adhesins have a similar structural organization to LapA and LapF and are widespread throughout prokaryotes suggesting that a similar mechanism for biofilm formation exists (Lasa and Penadés, 2006; Yousef and Espinosa‐Urgel, 2007).

### Plant–bacteria interactions influence colonization

Plant roots grow among diverse bacterial communities with up to 10^4^ bacterial species and 10^9^ bacterial cells per gram of soil (Daniel, 2005). Not surprisingly, the reservoir of bacteria in the surrounding soil is a crucial factor influencing root‐associated microbiome structure, as is illustrated by microbiome analyses of 27 inbred field‐grown maize lines planted at five geographically distinct locations across the United States (Peiffer *et al*., 2013). Albeit, plants do have some control over the composition of their root‐associated microbiome through modulation of their root exudate composition and mounting of an innate immune response (Lebeis *et al*., 2015; Reinhold‐Hurek *et al*., 2015; Sasse *et al*., 2018). As such, there are clear taxonomic differences between microbiota associated with the root and surrounding soil (Peiffer *et al*., 2013; Bulgarelli *et al*., 2015; Edwards *et al*., 2015; de Souza *et al*., 2016; Cheng *et al*., 2020). In *Medicago truncatula*, the rhizosphere and rhizoplane community structures become distinct after only 1 week of growth in soil. The community structure of both environments becomes more robustly established by the second week and remains stable for a minimum of 3 weeks thereafter (Tkacz *et al*., 2020). Crucially, microbiome structure varies across different plant species and even among genotypes within a single species (Kuske *et al*., 2002; Aira *et al*., 2010).

Plant root exudates contain a wide range of primary metabolites including carbohydrates, organic acids and amino acids, which preferentially stimulate bacterial growth thereby shaping microbiome assembly (Sasse *et al*., 2018). In addition, secondary metabolites present in root exudates can have antimicrobial activity against pathogens (Olanrewaju *et al*., 2019), act as signals for the establishment of root symbioses (Abdel‐Lateif *et al*., 2012) and have profound effects on the bacterial transcriptome (Ramachandran *et al*., 2011; Carvalhais *et al*., 2013). Root exudate composition varies between plant species and is dynamically influenced by developmental stage, environmental conditions and the structure of root‐associated microbiome (Sasse *et al*., 2018). Root colonization by specific microbial communities affects the chemical composition of root exudates through a systemic root‐to‐root signalling mechanism termed systemically induced root exudation of metabolites (SIREM) (Korenblum *et al*., 2020). In tomato split root assays, inoculation of *B*. *subtilis* onto one side of the root induced a systemic signal that results in increased secretion of acyl sugars from the uninoculated side of the root. Transportation of these signals through shoots to uncolonised areas of the root can modulate colonization and assembly of SIREM‐specific microbial communities.

The evidence supporting plant immune signalling as a regulator of root‐associated microbiome structure is less compelling but nevertheless important (Yu *et al*., 2019). Mutants of *A*. *thaliana* impaired in salicylic acid (SA) mediated defence displayed distinct microbiome composition relative to wild‐type plants (Lebeis *et al*., 2015). However, SA‐dependent signalling was shown to have the largest impact on endophytic community structure with rhizosphere microbiome structure less affected. In wheat, activation of the jasmonic acid (JA) signalling pathway through application of exogenous JA was found to reduce the diversity of the endophytic microbiome but not rhizosphere microbiome (Liu *et al*., 2017). In contrast, two *Arabidopsis* mutants disrupted in JA‐dependent signalling showed distinct rhizosphere microbiome structures relative to the wild type, though this could be associated with the fact that their root exudation profiles were also affected (Carvalhais *et al*., 2015). Additionally, aboveground activation of the immune system by plant pathogens and insects has been shown to alter the rhizosphere microbiome structure of several plant species (Dudenhöffer *et al*., 2016; Kong *et al*., 2016; Berendsen *et al*., 2018; Yuan *et al*., 2018). Again, alteration of rhizosphere microbiome structure may be linked to alteration of root exudate composition in response to activation of the immune system (Yuan *et al*., 2018). These results indicate that the immune system can influence epiphytic colonization, but colonization of the rhizosphere is indirectly influenced through the modulation of root exudates in response to the mounting of an innate immune defence.

### Bacteria–bacteria interactions influence colonization

Interactions among bacterial communities can be complex, involving both cooperation and competition (Deines and Bosch, 2016). Cooperative interactions include processes such as metabolite exchanges (Zelezniak *et al*., 2015), whilst production of antimicrobial toxins and deployment of mechanical weapons are examples of competitive interactions (Granato *et al*., 2019). Notably, most experiments characterizing factors required for bacterial colonization have done so with single strains under sterile laboratory conditions. A major challenge in understanding bacterial assembly in root niches is to move from single‐species studies to those that encompass entire communities to comprehend how individuals interact with each other and their host plant. Due to the enormous complexity of root‐associated microbiomes it is extremely challenging to experimentally characterize the molecular mechanisms underlying PBIs and BBIs, and their effects on plant health in natural systems. To facilitate this, synthetic communities (SynComs) have been used to investigate the role of individual species and plant‐derived metabolites during colonization of root niches (Niu *et al*., 2017; Voges *et al*., 2019). Importantly, SynCom assembly is affected by bacterial interactions (Mee *et al*., 2014), host genotype (Bodenhausen *et al*., 2014; Lebeis *et al*., 2015) and niche specificities (Bai *et al*., 2015). The same factors are known to affect the assembly of natural microbial communities, validating the use of SynComs as simplified microbiomes.

The power of using SynComs to study PBIs and BBIs was recently demonstrated with a simplified community of seven bacterial strains isolated from maize roots (Niu *et al*., 2017). When inoculated together, all members colonized to form a stable community on maize roots that reduced the prevalence of seedling blight by delaying colonization of the fungal pathogen *Fusarium verticillioides*. Removal of one specific SynCom member led to the failure of the remaining members to form a stable community, resulting in competitive dominance by a single strain which in isolation was less capable of warding off the pathogen. This study highlights the significance of BBIs during colonization of root niches and demonstrates that fundamental ecological principles, such as the role of keystone species, can be preserved within simplified SynComs. Thus far, the selection of bacterial strains used in SynComs has been guided by studies that have analysed the species composition of the specific plant‐associated microbial community when grown in soil. Moving forward, it could be of great benefit to identify a SynCom that is stable among different plant species. This would provide a simple system for probing specific population‐level determinants of colonization that are common to diverse plants or unique to individual plant species. The prospect of a ‘universal SynCom’ is not unfeasible since a similar set of plant‐associated bacteria is seen across diverse plant species (Müller *et al*., 2016).

### Tools to investigate bacterial colonization of root niches

Traditional single‐isolate experimental approaches cannot unravel the population‐level dynamics of bacterial root colonization or the molecular mechanisms driving them. However, new tools have been developed for marking and monitoring bacterial strains. These include technologies such as engineered transposons for genomic integration of reporter genes (Schlechter *et al*., 2018) and microfluidic imaging platforms for dynamic mapping (Massalha *et al*., 2017; Aufrecht *et al*., 2018; Noirot‐Gros *et al*., 2020). Moreover, the development of strategies for genome‐wide transposon mutagenesis screens is providing unprecedented opportunities to identify population‐level genetic determinants of colonization (Cain *et al*., 2020). These technologies are discussed in the following subsections.

#### Marking bacteria to track and quantify colonization

Marking of bacterial strains with reporter genes exhibiting unique spectral or other visual properties has been widely used to study root colonization, enabling distinction between differentially labelled bacteria and roots. (Bloemberg *et al*., 2000; Stuurman *et al*., 2000; Lagendijk *et al*., 2010; Ramirez‐Mata *et al*., 2018). Reporter genes are commonly expressed in bacteria using broad‐host‐range plasmids, but they can also be integrated into the chromosome by several strategies including homologous recombination (Ledermann *et al*., 2015), CRISPR‐Cas9 (Wang *et al*., 2018) or transposase‐based systems (Schlechter *et al*., 2018). Stable integration into the chromosome has the advantage of reduced dosage effects and improved stability in the absence of selective pressure, both of which are useful for studies in the rhizosphere and rhizoplane. Transposon‐based systems for DNA integration vary in their host range and mechanism. Mini‐Tn*5*, for example, functions in a wide range of Gram‐negative bacteria where it randomly inserts into the genome (de Lorenzo *et al*., 1990; Reznikoff, 2008), whilst mini‐Tn*7* integrates at specific attachment (*attB*_*Tn7*_) sites located downstream of the highly conserved chromosomal *glmS* gene (Bao *et al*., 1991; Craig, 1991). Importantly, mini‐Tn*7* integration of reporter genes typically has no detrimental effect on bacterial growth or competitiveness (Enne *et al*., 2005). Moreover, *attB*_*Tn7*_ sites are prevalent in phylogenetically diverse species making it an attractive tool for differentially labelling diverse bacteria (Parks and Peters, 2007).

**Fig. 2:**
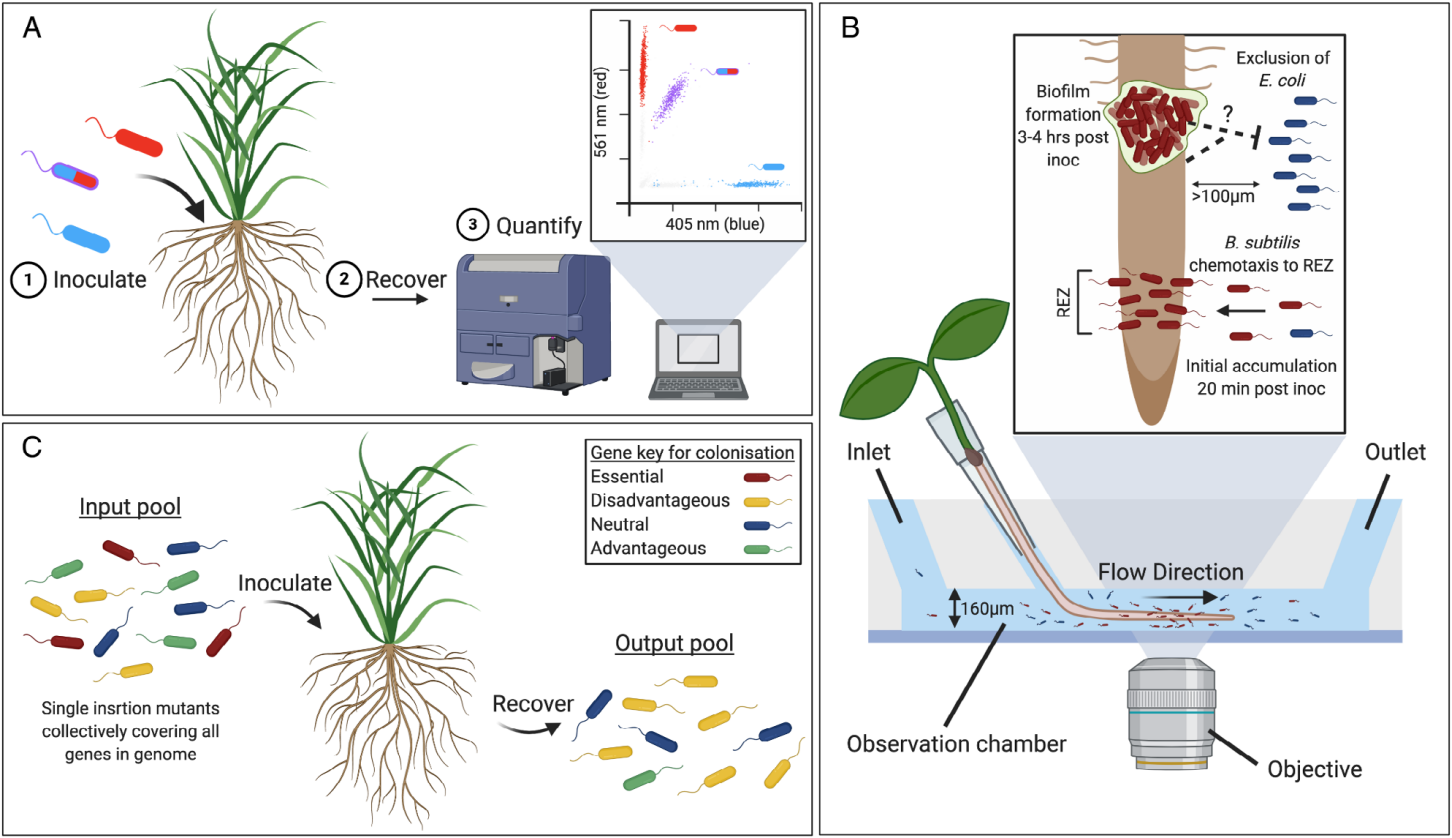
Tools to study bacterial root colonization. A. Flow cytometry: to track bacterial dynamics during colonisations bacterial species can be differentially marked with fluorescent proteins, e.g. red, blue or both, and quantified using flow cytometry. B. TRIS: a microfluidic device for real‐time visualization of bacterial–root interactions. The diagram shows a longitudinal section of a microfluidic channel containing root and bacterial cells (not drawn to scale). Seedlings are germinated through pipette tips into a channel to which bacteria can be introduced through the inlet. (Inset) a schematic of two bacteria, *B*. *subtilis* (red) and *E*. *coli* (blue) competing to attach to an *A*. *thaliana* root. TRIS showed that *B*. *subtilis* rapidly accumulates at the root elongation zone (REZ) within 20 min of bacterial inoculation, with subsequent aggregation occurring higher up the root (3–4 h post‐inoculation). *Escherichia coli* showed clear exclusion from the root, likely due to a diffusible element (represented by a dashed line) released by *B*. *subtilis* itself or the root when colonized by *B*. *subtilis* (Adapted from Massalha *et al*., 2017 ). C. Transposon mutagenesis screening: libraries containing single‐insertion transposon mutants that collectively cover all genes in the bacterial genome are inoculated onto a root system and recovered ‘X' days post‐inoculation. Comparison of input and output pools reveals whether a gene is essential (red), non‐essential (blue), advantageous (green) or disadvantages (yellow) for root colonization. Created with BioRender.com.

Marker genes such as *gusA*, *celB* and *lacZ* have traditionally been used to track bacteria on plants following incubation with a histochemical substrate, which the enzyme encoded by the reporter gene converts to a coloured product. (Sessitsch *et al*., 1998; Sánchez‐Cañizares and Palacios, 2013). Such reporters allow highly sensitive visualization of marked bacteria without the need for specialized equipment. However, in some instances, staining procedures result in significant cell death. Visualization of these markers is also affected by background activity in some bacteria and host plants (Sessitsch *et al*., 1998). More recently, fluorescent proteins have become the reporter of choice as they can be detected using non‐invasive methods allowing live cell imaging. There are many variants of fluorescent proteins that can be distinguished from one another based on their unique excitation and emissions wavelengths. By using fluorescent proteins with distinct spectral properties, a maximum of seven bacterial strains can be differentially marked and distinguished by confocal imaging (Schlechter *et al*., 2018). Fluorescent reporters can additionally be used for quantification of bacterial populations on the root surface by flow cytometry (Fig. 2A), which is a significantly more high‐throughput strategy than traditional culture‐dependent methods such as plate counting (Gamalero *et al*., 2004; Valdameri *et al*., 2015). Bioluminescent *lux*‐based reporters have also been used to measure bacterial attachment on whole‐root systems and for spatiotemporal mapping of root secretion (Pini *et al*., 2017). The primary benefit of using *lux* as a bioreporter over fluorescent proteins is improved sensitivity (Belkin, 2003). This is particularly useful when imaging bacteria during the early stages of root colonization, for example attachment at 1–2 h post‐inoculation of bacteria, since the number of cells on the root surface is relatively low (Parsons, 2019). However, light production via the *lux* proteins is energy‐intensive and can influence cell viability and competitiveness (Pini *et al*., 2017).

Competition between bacteria during colonization can alternatively be monitored by barcoding with oligonucleotides. In a recent example, 84 barcoded strains of *R*. *leguminosarum* were monitored for nodule occupancy of pea plants in co‐inoculation experiments (Mendoza‐Suárez *et al*., 2020). Sequencing of bacteria extracted from pea nodules was used to accurately determine the identity and relative abundance of rhizobial strains present in each nodule. In addition to carrying a unique barcode, each strain used in this study encoded a green fluorescent protein expressed from the promoter of the *nifHDK* (nitrogenase) operon, permitting crude quantification of N_2_ fixation. The ability to screen for elite competitiveness in large libraries of bacterial strains while simultaneously monitoring the expression of a biochemical marker will be revolutionary for the high‐throughput identification of agricultural inoculants.

### Imaging bacterial root interactions

Real‐time imaging of bacterial root interactions is particularly challenging since they occur belowground and vary drastically in spatial scale. To overcome this, plants may be grown in rhizotrons or cultured on agar plates or similar (Schmidt *et al*., 2011); however, these strategies are not amenable to the high‐resolution imaging techniques required for dynamic mapping of bacterial root interactions. The use of microfluidic platforms combined with live imaging microscopy provides the controlled conditions necessary for continuous imaging of bacterial root interactions at the cellular and subcellular resolution over several days (Massalha *et al*., 2017; Aufrecht *et al*., 2018; Noirot‐Gros *et al*., 2020). The microfluidic device tracking root interactions system (TRIS) consists of nine independent chambers through which roots can simultaneously be grown (Massalha *et al*., 2017). Fluorescently labelled bacteria can then be introduced into these chambers and imaged with confocal microscopy. TRIS revealed that distinct chemotaxis of *B*. *subtilis* towards the root elongation zone (REZ) of *Arabidopsis* preceded colonization over the entire root length. This indicates that the REZ is a hotspot for initial bacterial root interactions, likely due to high concentrations of root exudates and that bacterial chemotaxis and motility towards these exudates is a prerequisite for root colonization (Fig. 2B). Modification of the original TRIS device into a two‐channel system divided by a semipermeable membrane, that allows free movement of solutes and bacteria whilst preventing the roots of two plants from touching, enables real‐time tracking of bacterial preference for root genotypes (Massalha *et al*., 2017). One caveat of TRIS and similar devices is that the chamber width limits its use to plants with root diameters less than 160 μm. Consequently, observation of bacterial root interactions is limited to plants with narrow roots such as *Arabidopsis*. One exception is the root‐microbe interaction (RMI) chip, which facilitates the growth of roots up to 800 μm wide and was successfully used to study bacterial root interactions with Aspen and Rice seedlings (Noirot‐Gros *et al*., 2020). Adaption of these microfluidics platforms to facilitate the growth of larger roots, such as RMI‐chip, will allow investigation of bacterial root interactions in agronomically relevant crops including cereals and legumes.

Microfluidics platforms may also be used to study cooperative or competitive interactions between differentially marked bacteria. TRIS revealed that co‐inoculation of *B*. *subtilis* and *Escherichia coli* resulted in the exclusion of *E*. *coli* from *Arabidopsis* roots, indicating an antagonistic compound is released from either *B*. *subtilis* or roots colonized by *B*. *subtilis* (Fig. 2B) (Massalha *et al*., 2017). Moving forward with these technologies, extending the number of co‐inoculated bacterial species beyond two will provide invaluable insights regarding the influence of BBIs on the early stages of root‐niche colonization.

#### Transposon screening to identify genetic candidates

Whilst quantification and imaging of bacterial root colonization is a powerful technique for looking at physiology, alternative approaches are required to identify genetic determinants involved in bacterial colonization of roots. Transposon insertion sequencing (TIS) is one such approach. TIS is a powerful technique whereby libraries of single‐insertion transposon mutants, which collectively saturate an organism's genome, are exposed to a specific condition and then analysed with next‐generation sequencing to simultaneously estimate the essentiality and/or fitness contribution of each gene in a bacterial genome. Comparison of gene mutation frequency in an ‘input pool’ relative to an ‘output pool’ following a challenge such as root colonization reveals whether a gene is essential, non‐essential, advantageous, or disadvantageous for growth and survival (Fig. 2C). There are several variations of this technique including INSeq and TnSeq, each following these same basic principles, allowing the determination of gene fitness at the genome‐scale across a variety of conditions (Cain *et al*., 2020).

**Fig. 3:**
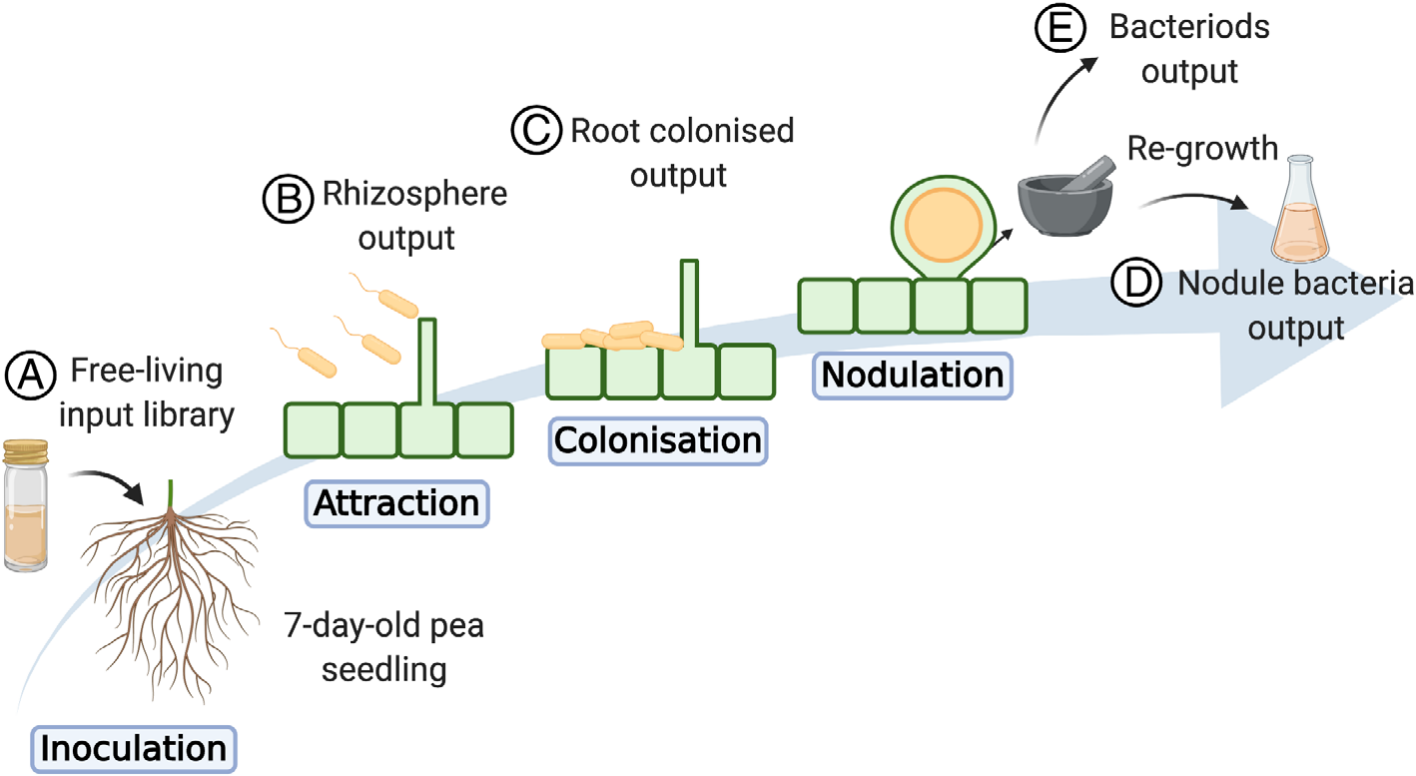
Lifestyle adaptations of Rhizobium from rhizosphere to symbiosis. Insertion sequencing was used to establish the role of *Rhizobium leguminosarum* bv. *viciae* 3841 (Rlv3841) genes at multiple stages of symbiosis with *Pisum sativum*. A. Rlv3841 transposon library was inoculated onto a 7‐day‐old pea seedling. Following inoculation bacteria were collected from four stages of symbiosis for analysis: (B) the rhizosphere (5 dpi), (C) the root (5 dpi), (D) nodule bacteria (28 dpi) and (E) N2 fixing bacteroides (28 dpi). Analysis of DNA purified from the input library and four output libraries enabled genome‐wide classification of gene fitness contributions at each stage. Created with BioRender.com.

Insertion Sequencing (INSeq) was recently used to identify bacterial genes important in the *Rhizobium*‐legume symbiosis at multiple stages of its development (Wheatley *et al*., 2020). To form a successful symbiosis, rhizobia must undergo several lifestyle changes. To investigate these, *R*. *leguminosarum* bv. *viciae* 3841 transposon libraries were inoculated onto its host legume *Pisum sativum* and recovered from four stages of the root‐nodule symbiosis: (i) free‐living growth in the rhizosphere, (ii) colonization of the root, (iii) nodule infection before differentiation into N_2_ fixing bacteroides and (iv) terminal differentiation into N_2_ fixing bacteroides (Fig. 3). While only 27 genes are assigned roles in the organization and regulation of N_2_ fixation, 593 genes were found to be required for the competitive ability to form a successful N_2_ fixing symbiosis. Of these, 146 were important for growth in the rhizosphere through to N_2_ fixing bacteroides, highlighting that competition in the rhizosphere is critical for establishing PBIs even in near‐isogenic populations.

A common limitation of most TIS techniques is that they require the mapping of transposon insertion locations for each mutant in the pool following exposure to each treatment. Random barcoded sequencing (RB‐TnSeq) is an extension of TIS techniques in which the transposable element contains a unique, but random, 20 nucleotide DNA ‘barcode’, so that each individual transposon mutant within a pool is identifiable by sequencing (Wetmore *et al*., 2015). After initial mapping of the transposon insertion site future experiments using the same mutant pool only require sequencing of the DNA barcodes in the input and output pools, saving considerable time and money and allowing multiplexing experiments where tens of strains can be simultaneously analysed. Such multiplexing experiments will be crucial for analysing how bacteria interact with the plant and one another during competitive root colonization. The power of RB‐TnSeq was recently demonstrated in a study that characterized mutant phenotypes of 32 diverse bacterial species in over 150 conditions to assign gene function *en masse*, resulting in the annotation of over 11 000 previously undefined protein‐coding genes (Price *et al*., 2018). Application of RB‐TnSeq to study *P*. *simiae* colonization of *A*. *thaliana* roots led to the identification of 115 genes required for optimal competitive colonization (Cole *et al*., 2017). These included genes with predicted roles in motility and carbon metabolism, and also 44 genes of unknown function. Undoubtedly, RB‐TnSeq experiments focussing on root colonization will continue to identify novel candidates involved in colonization. The next challenge will lie in deciphering their mechanism of action, particularly in the complex environment of the rhizosphere.

### Concluding remarks

Bacterial colonization of plant roots is a sequential, multi‐step process that begins in the rhizosphere with chemotaxis towards the root, followed by attachment and subsequent biofilm formation. To date, most genetic determinants involved in bacterial colonization have been evaluated in single‐strain studies under sterile conditions. Here we have highlighted that both PBIs and BBIs can influence bacterial colonization but characterizing their molecular mechanisms has been hindered due to the enormous scale of complexity and diversity of root‐associated microbiota. With the advent of new DNA‐integration systems and fluorescent reporters, marked seven‐member bacterial SynComs have been successfully cultivated offering unprecedented advancement towards deciphering the underlying principles of bacterial assembly in root niches. Moving forward, genetic exploration of SynComs using transposon mutagenesis‐based screening approaches will be instrumental in unveiling the novel genetics at play. Overall, a more thorough understanding of PBIs and BBIs will advance our understanding of bacterial ecological processes and be invaluable to optimize the future development of biofertilisers for sustainable agriculture, whether this be through targeted selection or engineering of elite plant growth‐promoting bacteria.
